# Development and validation of a short nutrition literacy scale for young adults

**DOI:** 10.3389/fnut.2023.1008971

**Published:** 2023-03-20

**Authors:** Jules Vrinten, Kathleen Van Royen, Sara Pabian, Charlotte De Backer, Christophe Matthys

**Affiliations:** ^1^Department of Chronic Diseases and Metabolism, KU Leuven, Leuven, Belgium; ^2^Department of Communication Sciences, University of Antwerp, Antwerp, Belgium; ^3^Karel de Grote University College, Research Centre The Cycle of Care, Antwerp, Belgium; ^4^Tilburg Centre for Cognition and Communication, Tilburg University, Tilburg, Netherlands; ^5^Department of Endocrinology, University Hospitals Leuven, Leuven, Belgium

**Keywords:** nutrition literacy, young adult, scale development, validation, surveys and questionnaires, health behavior, health literacy, nutrition

## Abstract

**Background:**

Due to their high media use, young adults are frequently exposed to contradictory or even erroneous nutrition information. To properly handle and critically assess nutrition information, young adults (both general population and patient populations) need adequate levels of nutrition literacy. Currently there is a lack of suitable instruments to measure nutrition literacy in young adults (18–25 years). Therefore the aim of this study was to develop and validate a Short Nutrition Literacy (S-NutLit) scale for use in this age group.

**Methods:**

Development and validation of the S-NutLit consisted of six phases: (1) item generation based on literature, (2) expert assessments to evaluate content validity, (3) cognitive interviews with the target population to assess face validity and readability, (4) pre-test to reduce the number of items, (5) validation survey to assess dimensionality with exploratory factor analyses, internal reliability with Cronbach alpha, construct and criterion validity by examining associations with age, gender, education level, health literacy, general literacy, dietary behaviors and physical activity with correlations, ANOVAs, and t-tests, (6) two-week follow-up survey to assess test–retest reliability with intra-class correlations.

**Results:**

Starting from an initial pool of 53 items, expert assessments and cognitive interviews led to the reformulation, removal, and construction of items. Young adults aged 18–25 years participated in cognitive interviews (*n* = 12), pre-test (*n* = 101), validation survey (*n* = 300), and reliability survey (*n* = 92). The final S-NutLit consisted of 11 items rated on a 5-point scale distributed across two subscales (i.e., information skills and expert skills). Cronbach alpha values ranged from 0.79 to 0.83 and intraclass correlations from 0.61–0.79 (*p* < 0.001). Significant associations were observed with health literacy (r = 0.27, *p* < 0.001), general literacy and numeracy (r_s_ = 0.12, *p* = 0.046), and education level (r_s_ = 0.13, *p* = 0.025).

**Conclusion:**

Findings indicate that the S-NutLit is a valid and reliable tool to assess nutrition literacy among young adults. The S-NutLit fills a gap in the field by offering a short measure of nutrition literacy and may be incorporated in digital technology to support the nutrition care process.

## 1. Introduction

Young adulthood is an important formative period in which lifelong health-related habits are established (1). Moreover, it is a period characterized by unhealthy dietary behaviors (2) and rapid weight gain (3), with all the adverse consequences this entails, such as type 2 diabetes, cardiovascular diseases, and several types of cancers (4, 5).

Concurrently, young adults display high levels of media use (6) and often turn to the Internet to seek nutrition information (7). This can make them susceptible to negative effects of misinformation and contradictory information regarding nutrition. Research has shown that online nutrition information can be contradictory and unreliable (8), which can result in feelings of confusion regarding nutrition (9). To properly handle and critically assess online nutrition information, young adults need adequate levels of nutrition literacy (NL).

Nutrition literacy is defined as the ability to obtain, process, and understand nutrition information to make appropriate nutrition-related decisions (10). Similarly to health literacy (11), NL can be subdivided into three domains: functional, interactive, and critical NL (12). Functional NL refers to basic literacy and numeracy skills necessary for obtaining and understanding nutrition information (13). For example, reading and understanding nutrition labelling and understanding dietary guidelines (14). Interactive NL refers to cognitive and interpersonal communication skills necessary for interactions with nutrition experts (12, 14). Additionally, interactive NL includes the ability to communicate and provide relevant nutrition information (15) and an interest in seeking and applying nutrition information (14). Critical NL refers to more complex cognitive and social skills necessary for the critical appraisal of nutrition information (14). Additionally, critical NL has an engagement dimension where people display willingness to participate in actions to remove socio-political barriers to healthy nutrition (e.g., campaigning for more affordable fruits and vegetables in school cafeterias) (14). Nutrition literacy has been associated with beneficial health outcomes and dietary behaviors in numerous studies among the general adult population (16–20) and among young adults (21–25).

There are several existing scales to measure NL such as the Nutrition Literacy Scale (NLS) (26), the NLit (27), and more recently the Young Adult Nutrition Literacy Tool (YA-NLT) (22). The NLS is a 28-item instrument modelled after the Short Test of Functional Health Literacy in Adults (S-TOFHLA) (26). In the NLS, respondents are shown sentences in which one or more words have been removed. Participants then choose the best fitting answer out of four options. The instrument is limited to functional NL by focusing on participants’ comprehension of nutrition information and nutrition knowledge and lacks assessment of skills in the interactive or critical NL domains (28). Another NL scale is the NLit (27). The NLit is an objective measure of NL and uses a multiple answer format to measure nutrition-related skills across six domains. The NLit was originally developed in the context of nutrition education and has subsequently been adapted and validated in other contexts and populations (16, 18, 29). An extended version with 64 items and a short version with 42 items are available. Even though the instrument is comprehensive in its assessment of nutrition knowledge and numeracy skills, the NLit lacks assessment of skills related to seeking, applying, and critically appraising nutrition information (28). A more recent attempt at comprehensively measuring NL can be found in the (22). The YA-NLT is a 42-item instrument that has been validated in a population of college students aged 18–24 years. Respondents are asked to self-assess their abilities in the domains of functional, interactive, and critical NL. Even though the YA-NLT assesses all three dimensions of NL, this instrument has been developed and validated in the US. Several items, especially of the functional NL subscale, are context-specific and not appropriate for the Belgian context. Moreover, the instrument is lengthy, making it less suitable in the context of monitoring and interventions. Therefore, the aim of this study was to develop a short NL scale (S-NutLit) that would cover all three domains of NL and to validate this in a population of Dutch-speaking young adults.

## 2. Materials and methods

**Figure 1:**
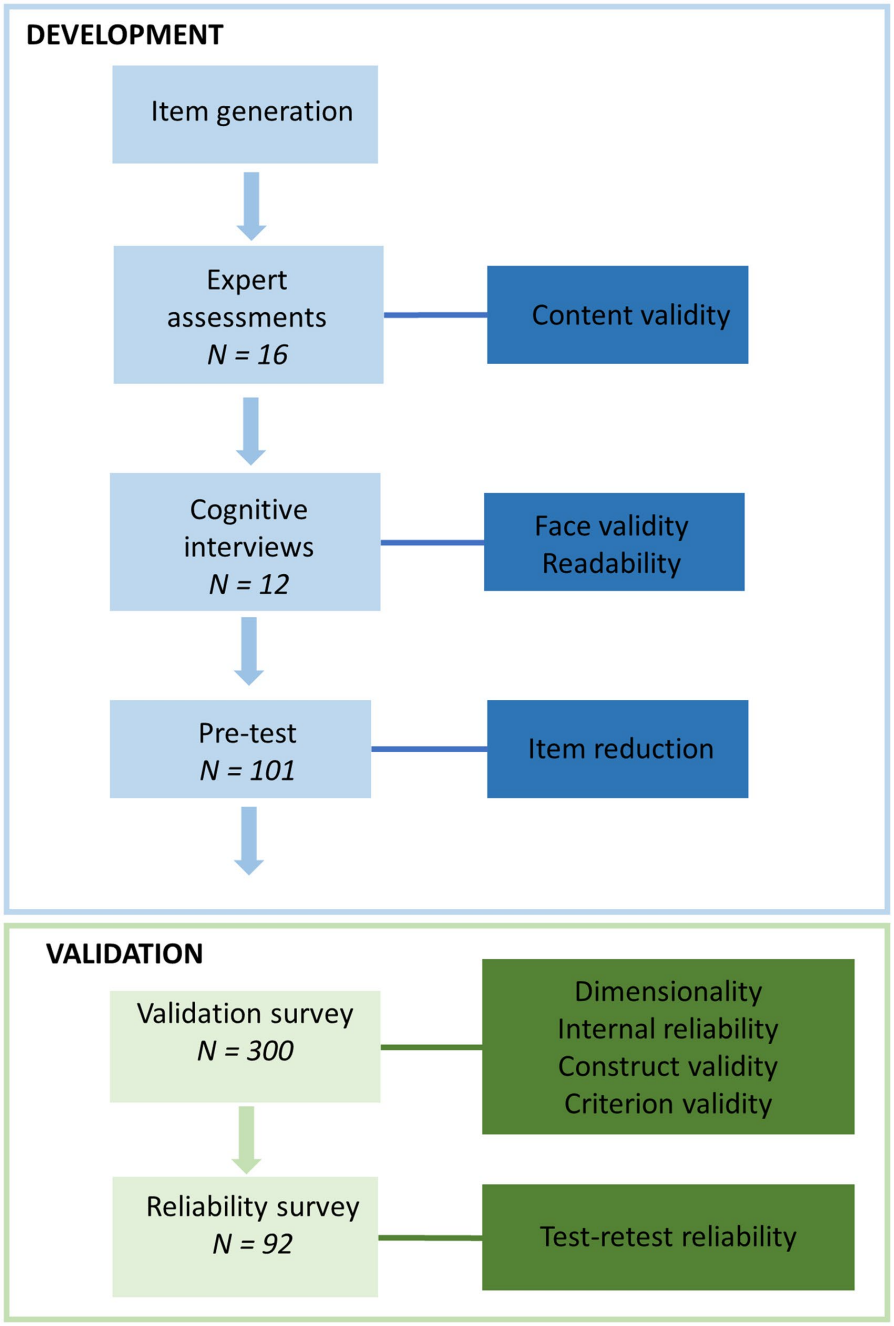
Steps in the scale development and validation process.

The development and validation of the S-NutLit followed a stepwise mixed method approach based on scale development methods by Polit and Beck (30) and Boateng (31) as stipulated in Figure 1. The different studies were conducted according to guidelines laid down in the Declaration of Helsinki. All procedures involving study participants were approved by the Ethics Committee for the Social Sciences and Humanities of the University of Antwerp (SHW_20_114, SHW_20_15).

### 2.1. Development of the S-NutLit

The development of the questionnaire consisted of the following steps: item generation, expert assessments, cognitive interviews, and pre-test.

#### 2.1.1. Item generation

Existing instruments in the domain of nutrition and health literacy were collected *via* Google Scholar and PubMed. Items from existing instruments were selected based on the extent to which they covered different aspects of NL. Several items were newly constructed (e.g., “I can evaluate whether nutrition advice from friends and family is reliable”) to allow differentiation between sources of nutrition information (e.g., family and friends, experts). Furthermore, several items pertaining to interactive NL (e.g., “I feel confident in using information from family members or friends to make nutrition-related decisions”) and sustainable (e.g., “I find it easy to find information on the social and environmental consequences (sustainability, desertification, equity …) related to my eating habits”) nutrition were added. Where necessary, items were rephrased to statements that could be rated on a 5-point Likert scale (1 = “*completely disagree*” to 5 = “*completely agree*”).

#### 2.1.2. Expert assessments

To assess content validity, experts in the field of nutrition, health promotion and health literacy evaluated the initial pool of items. The experts rated each item on a 4-point scale (1 = “*not relevant”* to 4 = “*highly relevant*”). An item content validity index was calculated as the proportion of experts rating an item as quite relevant or highly relevant (32). Different cut-offs were maintained for the item content validity index depending on the number of experts. With three to five experts, an item content validity index of 1 was considered adequate. With six to 10 experts, an item content validity index of 0.78 was the minimum. The scale content validity index was calculated as the average of all item content validity indices. A minimum scale content validity index of 0.90 was considered adequate.

#### 2.1.3. Cognitive interviews

To assess face validity and readability, cognitive interviews with the target population were conducted. A convenience sample of young adults was recruited *via* youth organizations, community health centers, and social media posts. Possible candidates were redirected to an online survey where their eligibility was assessed. Eligible candidates were invited for an interview. During the interviews, a probing technique was used to verify that participants understood formulation of the items (e.g., “*Can you explain in your own words what this question means?”* or “*What does this word mean according to you?”).* Concurrently, participants’ ability to work with the 5-point Likert rating scale response format was examined.

#### 2.1.4. Pre-test

To reduce the number of items, a cross-sectional survey was conducted among a convenience sample of university students. Participants were recruited *via* university staff members and internal communication channels. Inter-item correlations were used and a cut-off of 0.20 was maintained to discard highly uncorrelated items. Subsequently, principal component analysis with promax rotation was used to assess the potential need for discarding additional items. Promax rotation was used as it was expected that components would be correlated (33). Items were discarded if they failed to achieve a minimum of 0.50 for primary and a maximum of 0.20 for secondary component loadings (33). After the discarding of an item, principal component analyses were rerun and the necessity for discarding additional items was reviewed. This iterative process was continued until all items met the cut-offs of 0.50/0.20.

### 2.2. Validity and reliability of the S-NutLit

#### 2.2.1. Study design, setting and sample size

Two cross-sectional surveys were used to assess validity and reliability of the S-NutLit. Data were collected from January to February 2022 *via* an online survey on a panel of young adults aged 18 and 25 years. Participants were recruited *via* an external recruitment company. Stratified sampling was employed to ensure an even distribution of gender. Written consent was obtained from all participants at the start of the survey. Participants were considered eligible for participation if they were between 18 and 25 years of age and proficient in Dutch. Two weeks after the first survey, the S-NutLit was retaken by a subset of participants to assess test-rest reliability. To assess validity, a sample size of *n* = 300 was deemed sufficient (33). To assess test–retest reliability, a minimum sample size of *n* = 80 was deemed sufficient, which takes into account an expected drop-out rate of 20% (34).

#### 2.2.2. Measures

##### 2.2.2.1. Demographics

Participants were asked about their age, gender, and highest obtained education level. Additionally, participants were asked if they were studying/had obtained a degree in a health- or nutrition-related field (e.g., dietetics, nursing, medicine, dentistry). Frequency of receiving nutrition information from experts and from friends/family was assessed with two questions using a 5-point scale (1 “never” to 5 “always”).

##### 2.2.2.2. Health literacy

To assess subjective health literacy, a short form of the European Health Literacy Survey was employed (HLS-EU-Q16) (35). The HLS-EU-Q16 is a 16-item health literacy scale that assesses skills related to accessing, understanding, appraising, and applying health information in the context of health care, disease prevention, and health promotion (e.g., “*How easy or difficult is it for you to understand information in the media about how to become healthier?”)*. Answers were given on a 4-point scale (1 = “*very difficult*” to 4 = “*very easy*”) and subsequently dichotomized into 0 (“*very difficult*” and “*fairly difficult*”) and 1 (“*fairly easy*” and “*very easy*”) (36). Dichotomized scores were summed and used in further analyses.

##### 2.2.2.3. General literacy and numeracy

A validated Dutch version of the Newest Vital Sign (NVS-D) was used to assess general literacy and numeracy (37). The NVS-D consists of six open-ended items assessing comprehension of an ice cream nutrition label (e.g., “*If you eat the entire container* [of ice-cream]*, how many calories will you eat?*”). The NVS-D has an acceptable internal reliability (Cronbach’s alpha = 0.76).

##### 2.2.2.4. Dietary behavior

Dietary behavior was measured with items from a widely used Belgian population survey (38). Several items related to the frequency and quantity of the consumption of fruit (e.g., “*How often do you consume fruit, not including juice from fresh fruit or from concentrate?”)*, vegetables (e.g., “*How many servings of vegetables or salad, excluding juice and potatoes, do you eat each day?”),* sugar-sweetened beverages (e.g., *“How often do you drink sugar-sweetened soft drinks, such as lemonade, cola or iced tea?”*), snacks (“*How often do you eat sweet or salty snacks such as candy, chocolate, pastries, cookies, ice cream, chips,…?*”), and juice (“*How often do you drink 100% pure fruit or vegetable juice, excluding juice prepared from concentrate and sugar-sweetened juices?*”). Due to the focus on NL, only a short assessment of dietary behavior was made though this can be considered sufficient given the importance and predictive validity of individual dietary behaviors (39).

##### 2.2.2.5. Physical activity

To assess physical activity the short version of the International Physical Activity Questionnaire (IPAQ-SF) was used (40). The IPAQ-SF is a 7-item instrument that measures physical activity in four domains over the last 7 days (e.g., “*Thinking about the past 7 days, on how many of these days did you perform vigorous physical activities such as lifting heavy loads, digging, aerobics or cycling?*”). The IPAQ-SF is a widely used instrument that is as valid and reliable as the IPAQ long form (40) but more user friendly (41). The official scoring protocol was followed for the data cleaning and analysis of the IPAQ data (42).

#### 2.2.3. Statistical analyses

Data from the validation survey were subject to analyses related to dimensionality, internal reliability, and validity. Data from the reliability survey were used to assess test–retest reliability.

##### 2.2.3.1. Dimensionality

We aimed to investigate whether the three dimensions of NL (functional, interactive, and critical) could be derived from the data by exploring the underlying structure of latent factors. Therefore, Exploratory Factor Analyses (EFA) with promax rotation were used (33). Sampling adequacy was assessed by the Kaiser-Meyer-Olkin measure (KMO = 0.85) and Bartlett’s test of sphericity. The number of factors was determined by visual inspection of the scree plots and the eigenvalues (minimum of 1). For the factor loadings, both the pattern and structure matrices were observed and cut-offs of minimum 0.50 for primary and maximum 0.20 for secondary loadings were maintained (33). If an item failed to meet these criteria, the item was discarded and the EFA was repeated. This process was repeated until all items met the criteria.

##### 2.2.3.2. Internal reliability

Cronbach’s alpha was used to assess internal reliability. An alpha of 0.70 or higher was considered acceptable (43).

##### 2.2.3.3. Validity

To assess convergent construct validity, a mean score of the S-NutLit was calculated and associations with related constructs were observed. Pearson correlations were used for continuous variables and ANOVA’s and independent samples *t*-tests for mixed data. Spearman correlations were used where the assumption of normality was not met, based on visual inspection of histograms and P–P plots. Correlations of 0.10 were considered small, 0.30 moderate, and 0.50 large (44). It was expected that related constructs (e.g., health literacy, general literacy) were positively associated with the S-NutLit providing evidence for convergent validity. To assess criterion validity, associations with behaviors (i.e., dietary behaviors and physical activity), were examined.

##### 2.2.3.4. Test–retest reliability

Data from the final survey were used to assess test–retest reliability. Intraclass correlations (ICC) were used to assess test–retest reliability (45). An ICC of less than 0.50 was considered poor, between 0.50 and 0.75 moderate, between 0.75 and 0.90 good, and greater than 0.90 excellent (46). All statistical analyses were conducted in SPSS version 28.0 (IBM Corp, 2021, Armonk, NY, United States).

## 3. Results

### 3.1. Development of the S-NutLit

#### 3.1.1. Item generation

Initial item generation was based on existing instruments (14, 36, 47–53), and where necessary, new items were constructed resulting in a preliminary pool of 53 items. Items from existing instruments were translated backwards with the help of a native English speaker. Of the 53 items, 32 were taken verbatim from existing instruments, 11 were modified from existing instruments, and 10 were newly constructed. Six of 10 new items were related to different sources of nutrition information, three to interactive NL, and one to sustainable nutrition. All items were rated on a 5-point scale ranging from 1 “*completely disagree*” to 5 “*completely agree*.” Higher scores indicated a higher level of NL.

#### 3.1.2. Expert assessments

For content validity, the 53 items were assessed by experts in the field of nutrition, health promotion and health literacy (*n* = 16). After two rounds of expert assessments, a total of 48 items with a scale content validity index of 0.90 remained. Wording of items was modified based on expert advice.

#### 3.1.3. Cognitive interviews

Face validity and readability were assessed by cognitive interviews with a group of young adults aged 20–25 years (*n* = 12, mean age 22.6 (*SD* = 2.1), 50% women). Based on the interviews, several unclear and problematic items were discarded. For example, items that related to attitudes rather than skills were removed (e.g., “*I think it is important that information about nutrition is based on scientific research*”). Other items were removed because it was unclear whether a high score on these items could be considered favorable (e.g., “*I am critical of the nutrition information I receive from experts*”). Several items were reformulated to increase readability. Specifically, items were shortened, unnecessary clauses were removed, difficult terminology was substituted, and items were reworded to bring them more in line with other items. For example, the item “*I am able to decide when a claim about how healthy something is, is based on scientific research and when it is not*” was shortened to *“I can judge when a claim about how healthy something is, is based on scientific research.”* Additionally, a language expert reviewed the readability of the items and small modifications were made to further enhance readability. Moreover, several changes were made to the answer options. Wording of the central answer option was changed from “*neither agree nor disagree*” to “*neutral*.” Furthermore, a “not applicable” option was added to one item (“*I have the necessary skills to apply nutrition information when cooking*”) as participants indicated that this item could be irrelevant to those that do not cook. The answer options of several items (items 1 and 9–11) were changed to 1 = “*never*” to 5 = “*always*” as this provided a better match with the content of the items. After the interviews, the initial version of the S-NutLit consisted of 28 items.

#### 3.1.4. Pre-test

To reduce the number of items, the 28-item S-NutLit was pre-tested in a convenience sample of university students (*n* = 101, mean age = 21.7 (*SD* = 3.8), 85.1% women). Inspection of the inter-item correlations led to the initial discarding of four items. Inspection of the pattern and structure matrices of the principal component analyses led to the discarding of an additional 12 resulting in a final set of 12 items.

### 3.2. Validity and reliability of the S-NutLit

#### 3.2.1. Participant characteristics

Characteristics of participants in the validation and reliability surveys are displayed in Table 1. In a first survey, the validity of the S-NutLit was assessed on a stratified sample [*n* = 300, mean age = 21.6 (*SD* = 2.2), 53% women]. Participants in the first survey were primarily higher educated with 49.3% of participants having completed tertiary education. Overall, 28.0% of participants were active in a health- or nutrition-related field. In a second survey, the reliability of the S-NutLit was assessed [*n* = 92, mean age = 22.0 (*SD* = 2.1), 67.4% women]. The percentage of women and the average age were slightly higher than in the first survey. The distribution of education level and the percentage of participants active in a health-related field were comparable.

**Table 1:** Demographic characteristics of participants in the validation and reliability survey.

	Validation survey *N* = 300	Reliability survey *N* = 92
Characteristics	*N* (%)/Mean (*SD*)	*N* (%)/Mean (*SD*)
Gender		
Women	159 (53.00)	62 (67.39)
Men	139 (46.33)	30 (32.61)
Non-binary	2 (0.67)	0 (0)
Age, y	21.6 (2.20)	22.0 (2.07)
Education level		
Primary	12 (4.00)	1 (1.09)
Secondary	140 (46.67)	40 (43.48)
Tertiary	148 (49.33)	51 (55.43)
Active in a health-related field	84 (28.00)	24 (26.09)

#### 3.2.2. Dimensionality

To explore dimensionality of the S-NutLit, Exploratory Factor Analyses with promax rotation were conducted. Sampling adequacy was confirmed based on the Kaiser-Meyer-Olkin measure (KMO = 0.85) and Bartlett’s test of sphericity (χ^2^ = 1122.53, df = 66, *p* < 0.001). Based on both the eigenvalues (minimum of 1) and a visual inspection of the scree plot, two factors were retained. An inspection of the pattern and structure matrices indicated that one item (“*I can judge when a claim about how healthy something is, is based on scientific research*”) did not meet the predetermined cut-off of 0.50/0.20, hence this item was discarded. Subsequently, the EFA was repeated with the remaining 11 items. Again, the Kaiser-Meyer-Olkin measure (KMO = 0.83) and Bartlett’s test of sphericity (χ^2^ = 970.27 df = 55, *p* < 0.001) confirmed sampling adequacy. In this second EFA, all items were retained. The final 11 items with their factor loadings in the pattern matrix are presented in Table 2. Two subscales were distinguished that explained 44.3% of total variance. The first subscale consisted of eight items and focused on information skills (e.g., seeking, applying, and appraising nutrition information). The second subscale consisted of three items and focused on the use of experts and scientific knowledge. The subscales displayed a correlation of r = 0.20, *p* < 0.001.

**Table 2:** Pattern matrix of the exploratory factor analysis.

Items		Factor loadings	
		Information skills (0.83)	Expert skills (0.79)
1	I can assess whether information about nutrition in the media is reliable.	0.79	−0.04
2	If I have questions about healthy nutrition, I know where to find information about it.	0.69	−0.06
3	When searching for nutrition information on the Internet, I can distinguish between reliable and less reliable websites.	0.62	−0.02
4	If I have questions about sustainable nutrition, I know where to find information about it. Examples of sustainable nutrition are organic vegetables, free-range chicken eggs, fair trade coffee, etc.	0.61	−0.01
5	I have the necessary skills to apply nutrition information when cooking.	0.58	0.03
6	Advertisements often make a connection between nutrition and health. I find it easy to judge whether these links are true or not.	0.58	0.14
7	I know the basic rules of the Flemish Food Triangle.^1^	0.57	0.01
8	I can assess whether information about nutrition is written with the intention of making money, for example by people who want to sell a product.	0.54	0.03
9	I follow nutrition advice from experts.	−0.08	0.91
10	I discuss nutrition information with experts.	0.03	0.71
11	I base my diet on the latest scientific knowledge.	0.09	0.61

1 The Flemish Food Triangle is an educational model of an inverted pyramid depicting dietary guidelines.

#### 3.2.3. Internal reliability

For the entire S-NutLit Cronbach’s alpha was 0.80. For the subscale “information skills” Cronbach’s alpha was 0.83 and for the subscale “expert skills” 0.79. Mean scores for the final 11 items and for the two separate subscales were calculated and used in subsequent analyses. Mean scores were 3.25 (*SD* = 0.59), 3.52 (*SD* = 0.65), and 2.55 (*SD* = 1.00) for the S-NutLit, the “information skills” subscale, and the “expert skills” subscale, respectively.

#### 3.2.4. Validity

To assess convergent construct validity, associations with several constructs were observed (Table 3). A moderate, positive association was observed with health literacy (r = 0.27, *p* < 0.001). This association was slightly stronger for the subscale “information skills” (r = 0.40, *p* < 0.001), but not significant for the “expert skills” subscale (r = −0.10, *p* = 0.094). Additionally, a small, positive association was observed with general literacy and numeracy (r_S_ = 0.12, *p* = 0.046). This association was slightly stronger for the subscales, but the pattern was dissimilar; there was a positive association with the “information skills” subscale (r_S_ = 0.27, *p* < 0.001), but a negative association with the “expert skills” subscale (r_s_ = −0.26, *p* < 0.001). The association with education level was small for both the S-NutLit (r_s_ = 0.13, *p* = 0.025) and for the “expert skills” subscale (r_s_ = 0.16, *p* = 0.007). No significant gender differences were found except for the subscale “expert skills,” with men scoring significantly higher than women [*t* (287.955) = 2.42, *p* = 0.008; Table 4]. Participants active in a health- or nutrition-related field scored significantly higher on the S-NutLit and the two subscales. Frequency of receiving nutrition information from both experts and friends/family was positively associated with the S-NutLit. Especially the association between exposure to nutrition information from experts and the “expert skills” subscale was strong (r = 0.48, *p* < 0.001). Associations with age were non-significant.

**Table 3:** Associations between S-NutLit, subscales and related constructs.

	S-NutLit	Information skills	Expert skills
Spearman correlations			
Education level	0.13*	0.06	0.16*
General literacy and numeracy	0.12*	0.27**	−0.26**
Vigorous physical activity^1^	−0.18*	−0.24*	0.07
Moderate physical activity^1^	−0.01	−0.16	0.28**
Walking^1^	0.03	−0.07	0.21*
Sitting^1^	−0.01	0.16	−0.28*
Fruit^2^	0.08	0.02	0.16*
Vegetables^2^	0.05	0.11	−0.06
Juice^2^	0.08	−0.08	0.32**
Sugar-sweetened beverages^2^	0.15*	0.15*	0.04
Snacks^2^	0.05	−0.07	0.20**
Pearson correlations			
Age	0.02	0.02	0
Health literacy	0.27**	0.40**	−0.10
Nutrition information from experts	0.28**	0.07	0.48**
Nutrition information from friends/family	0.28**	0.17*	0.32**

* Significant at *p* < 0.05.

** Significant at *p* < 0.01.

1 Minutes per day.

2 Portions per week.

**Table 4:** T-tests of S-NutLit and the two subscales for gender and participants active in a health-related field.

	S-NutLit		Information skills		Expert skills	
Gender^1^	*t*(270.41) = 1.00	*p* = 0.160	*t*(272.774) = −0.12	*p* = 0.451	*t*(287.955) = 2.42	*p* = 0.008*
Active in a health-related field^2^	*t*(140.263) = −4.43	*p* < 0.001**	*t*(137.984) = −2.09	*p* = 0.019*	*t*(169.160) = −6.48	*p* < 0.001**

* Significant at *p* < 0.05.

** Significant at *p* < 0.01.

1 Men (*n* = 139) vs. women (*n* = 161).

2 No (*n* = 216) vs. yes (*n* = 84).

To assess criterion validity, associations with various behaviors were examined. The pattern of associations with dietary behaviors was diverse with several small associations and only one moderate association between juice consumption and the “expert skills” subscale (r_s_ = 0.32, *p* < 0.001). Noteworthy are the unexpected positive associations between the consumption of sugar-sweetened beverages and the S-NutLit. The pattern of associations with physical activity displayed equal diversity with several expected and unexpected findings. Favorable, expected associations were found between the “expert skills” subscale and moderate physical activity, walking, and sitting. Unexpected associations were observed between vigorous physical activity and the S-NutLit and “information skills” subscale.

#### 3.2.5. Test–retest reliability

The two-way mixed ICC for the S-NutLit was 0.74 (95% CI [0.61, 0.83], *p* < 0.001). The ICC for the two subscales of the S-NutLit were 0.79 (95% CI [0.69, 0.86], *p* < 0.001) and 0.61 (95% CI [0.42, 0.75], *p* < 0.001) for the subscales “information skills” and “expert skills,” respectively.

## 4. Discussion

The aim of the current study was to develop and validate a short NL scale for young adults (18–25 years). A rigorous, step-by-step approach, which consisted of expert assessments, cognitive interviews, and three separate surveys, was followed. Novel in the validation process were the associations with validated measures of health literacy, general literacy, and physical activity. The S-NutLit was proven to be a valid and reliable tool and fills a gap in the field by offering a short, yet comprehensive measure of NL. The brevity of the S-NutLit makes it ideally suited for incorporation into digital technology to support the nutrition care process.

Construct validity of the S-NutLit was supported in several ways. First, a significant association between health literacy and the S-NutLit was found. This finding is unsurprising given the conceptual overlap between nutrition and health literacy (10, 54). Other studies in the domain of NL and nutrition-specific health literacy have provided inconsistent findings with studies reporting no significant association (16) or slightly stronger associations (29, 47). Inconsistent findings might be attributed to the heterogeneity of measurement instruments in the domain of health and NL. Second, a significant positive association was observed between the S-NutLit and education level of the young adult. This is in line with findings from previous research demonstrating that lower NL is associated with lower levels of education (17, 55–57) and points to the relevance of education as a determinant of NL (58). Third, individuals active in a health- or nutrition-related field scored consistently higher on the S-NutLit and the two subscales. This provides evidence for convergent validity since this association could be expected based on earlier findings (57). Lastly, the S-NutLit was significantly associated with frequency of receiving nutrition information from experts and from friends/family. This finding is in line with prior research highlighting the positive influence of a person’s environment on NL (59) and nutrition knowledge (60). In addition to sufficient construct validity, the S-NutLit and its subscales also displayed acceptable internal reliability and moderate to good test–retest reliability, the magnitudes of which are comparable to that of existing scales in the field (14, 19, 22, 26). The ICC for the “expert subscale” was somewhat lower than that of the S-NutLit or the “information skills” subscale. However, the confidence interval for the ICC of the “expert skills” subscale was also considerably larger. It is thus possible that the estimate of the ICC for the “expert skills” subscale is less accurate than those of the S-NutLit or the “information skills” subscale.

Criterion validity of the S-NutLit and its subscales was less well supported. Only the “expert skills” subscale showed signs of criterion validity by exhibiting favorable associations with different dietary and physical activity behaviors. For example, the “expert skills” subscale was significantly, positively associated with moderate physical activity, walking, fruit consumption, and juice consumption. However, the “expert skills” subscale was also significantly, positively associated with sedentary behavior and snack consumption. Associations with S-NutLit and the “information skills” subscale were primarily non-significant or even contrary to expectations. For example, a positive association with the consumption of fruits and vegetables was expected yet not found (16). Additionally, an unexpected association was observed where participants with higher levels of NL reported a greater consumption of sugar-sweetened beverages. Furthermore, significant negative associations were observed with vigorous physical activity. These mixed findings are in line with previous studies that reported differential associations between (subscales of) NL instruments and dietary indices and/or single dietary indicators (16–20). Whereas most studies in the NL domain have employed dietary indices, there is some debate regarding the predictive validity of dietary indices versus single dietary indicators (39). Furthermore, not all dietary indices are equally valid (61). Further research is warranted to explore associations with dietary behavior bearing in mind the limitations of dietary indices and the differential predictive validity of these indices.

Several expected associations were not observed. For example, it was expected that women would score higher on the S-NutLit (21, 47), yet no significant gender differences were observed except for the subscale “expert skills” where men scored significantly higher than women. Post-hoc independent samples t-tests revealed that men in our sample more frequently received nutrition information from experts than women. Prior research has shown that men have a pronounced preference for experts such as dietitians (62). However, other studies have found no significant gender differences regarding the preference of dietitians as source of nutrition information (63–65) or even found the reverse to be true (66). Further exploration of potential gender differences in the use of and preference for expert sources of nutrition information is warranted. Furthermore, contrary to findings in previous studies (17, 56), there was no significant association with age. This may be explained by the limited range of age in this sample. A limited data range suppresses variability, which in turn influences potential associations (67).

The development and validation process resulted in a final version of the S-NutLit containing 11 items divided across two subscales, information skills and expert skills. Contrary to expectations, the three dimensions of NL were not distinguished as factors in the exploratory factor analyses. Instead, only two subscales were withheld with all items relating to functional and critical NL loading unto the subscale “information skills.” The “expert skills” subscale consisted of items related to interactive NL and more specifically the use of experts and scientific knowledge. Only one interactive NL item (“*I have the necessary skills to apply nutrition information when cooking*”) loaded unto the “information skills” subscale. The intricate complex relationships between functional, interactive, and critical NL have surfaced in previous research (22, 53). For example, in their development and validation study, McNamara et al. (22) conducted separate factor analyses for the three dimensions of NL and found several factors within those dimensions. Specifically, two factors were distinguished within their functional subscale and four factors within their critical NL subscale. Thus, it appears that the tripartite of functional, interactive, and critical NL is less straightforward than it seems, and that further research is warranted to explore the dimensionality of the NL construct.

The distinction between skills related to nutrition information on the one hand and the use of experts and scientific knowledge on the other hand was further emphasized by differential patterns of associations. For example, the S-NutLit and “information skills” subscale were positively associated with general literacy and numeracy whereas the “expert skills” subscale was not. Additionally, the “expert skills” subscale displayed a favorable pattern of associations with several physical activity indicators, whereas associations with the S-NutLit and the “information skills” subscale were non-significant or even contrary to expectations. Moreover, the “expert skills” subscale displayed a greater number of significant associations than the S-NutLit and the “information skills” subscale. This may be explained by a difference in variability. The standard deviation of the “expert skills” subscale was substantially larger than that of the S-NutLit and of the “information skills” subscale. As was the case with age, limited variability may inhibit potential associations (67). Given the conciseness of the “experts skills” subscale and the favorable pattern of associations, measuring skills related to the use of experts and scientific knowledge may be of added value in the assessment of NL.

### 4.1. Strengths and limitations

As many studies, the current study is confronted with limitations. First, despite best efforts to reach young adults from a diverse range of socioeconomic backgrounds, the samples used to develop and validate the S-NutLit were biased towards higher socioeconomic status (SES). This may impede the validity and applicability of this scale in populations of young adults of lower SES. Recruitment for the cognitive interviews was ongoing during the COVID-19 pandemic when most organizations working with people from lower SES had other priorities. This impeded the inclusion of young adults from lower SES. Recruitment for the validity and reliability surveys was conducted by an external recruitment company, which despite best efforts equally struggled to recruit young adults of lower SES. Due to practical considerations, it was not possible to devote additional efforts to recruiting young adults of lower SES. Second, the sample size for the pre-test was too small for principal component analyses, which may have led to an excessive discarding of items. Larger sample sizes are recommended for factor structures that are less easily replicable, especially when there are less than five items per factor (33, 68). Third, many participants in the validation study indicated that they were less physically active and more sedentary due to the exam period during which data was collected. This may have distorted answers on the IPAQ. Additionally, inspection of the distribution of the IPAQ data revealed ceiling effects. This is a direct consequence of following the official IPAQ scoring protocol and points to limitations of the scoring protocol. Fourth, statistical indicators were prioritized in the discarding of items. By overly relying on statistical indicators, there is a danger that the entire breadth of a domain is not adequately covered since items that are strongly related are more likely to be selected (69). For example, items related to the engagement dimension of critical NL were discarded due to weak associations with other items. It is recognized that due to the limited research in the field of NL, the concept of NL lacks focus and conceptual clarity (70). Not surprisingly, this lack of conceptual clarity is particularly apparent in the development of operational tools. Despite its limitations, the current study also features several strengths. First, a rigorous stepwise approach was followed that is in line with current recommendations on scale development (30, 31). Second, contrary to prior scale development efforts in the field of NL, validity was assessed by observing associations with validated and widely used measures of health literacy, general literacy, and physical activity. Third, due to the inclusion of different groups throughout the scale development process (i.e., not only experts in nutrition and health literacy but also members of the target group) the S-NutLit has been developed from a variety of viewpoints further contributing to its validity.

### 4.2. Implications for research and practice

Researchers and health promotion professionals can use the S-NutLit to monitor NL in young adults. Due to its brevity, the S-NutLit can be incorporated into existing instruments aimed at assessing dietary behavior (e.g., the Belgian Food Consumption Survey (71)). Monitoring of NL in young adults can inform the development of broader interventions that are not limited to disseminating nutrition knowledge, as is often the case (72), but also on skills related to coping with mediated nutrition information. Furthermore, short instruments like the S-NutLit are ideally suited for inclusion in eHealth or mHealth interventions. Future studies could include additional validation in other and more diverse samples of young adults. Especially populations of young adults of lower SES should be included. Moreover, future studies are warranted to assess the sensitivity of the S-NutLit and to determine cut-off scores to distinguish between those with high and low NL.

## Data availability statement

The raw data supporting the conclusions of this article will be made available by the authors, without undue reservation.

## Ethics statement

The studies involving human participants were reviewed and approved by Ethics Committee for the Social Sciences and Humanities of the University of Antwerp (SHW_20_114, SHW_20_15). The patients/participants provided their written informed consent to participate in this study.

## Author contributions

CB, CM, and JV: conceptualization. JV, SP, KR, CB, and CM: methodology. JV: data collection, data analysis, and writing – original draft preparation. JV, SP, KR, and CM: writing – reviewing and editing. CB, KR, and CM: supervision. CB and CM: funding acquisition. All authors contributed to the article and approved the submitted version.

## Funding

This work was supported by the Flanders Innovation & Entrepreneurship and Flanders’ FOOD under Grant (HBC.2018.0397).

## Conflict of interest

The authors declare that the research was conducted in the absence of any commercial or financial relationships that could be construed as a potential conflict of interest.

## Publisher’s note

All claims expressed in this article are solely those of the authors and do not necessarily represent those of their affiliated organizations, or those of the publisher, the editors and the reviewers. Any product that may be evaluated in this article, or claim that may be made by its manufacturer, is not guaranteed or endorsed by the publisher.

## Abbreviations

ANOVA: Analysis of variance
EFA: Exploratory factor analysis
ICC: Intraclass correlations
HLS-EU-Q16: European health literacy survey was employed
IPAQ: International physical activity questionnaire
IPAQ-SF: International physical activity questionnaire – short form
KMO: Kaiser-Meyer-Olkin
NL: Nutrition literacy
NLS: Nutrition literacy scale
NVS-D: Dutch version of the newest vital sign
SD: Standard deviation
SES: Socioeconomic status
S-NutLit: Short nutrition literacy scale
S-TOFHLA: Short test of functional health literacy in adults
YA-NLT: Young adult nutrition literacy tool
